# Dissolving Microneedles for Intradermal Vaccination against Shigellosis

**DOI:** 10.3390/vaccines7040159

**Published:** 2019-10-24

**Authors:** Yadira Pastor, Eneko Larrañeta, Álvaro Erhard, Gemma Quincoces, Iván Peñuelas, Juan M. Irache, Ryan Donnelly, Carlos Gamazo

**Affiliations:** 1 Department of Microbiology and Parasitology, Institute of Tropical Health, University of Navarra, 31008 Pamplona, Spain; ypastor@alumni.unav.es; 2 Institute of Tropical Health, University of Navarra, 31008 Pamplona, Spain; jmirache@unav.es; 3 School of Pharmacy, Queen’s University Belfast, Medical Biology Centre, 97 Lisburn Road, Northern Ireland, Belfast BT9 7BL, UK; e.larraneta@qub.ac.uk (E.L.); aerhard@unav.es (Á.E.); 4 Medical Biology Centre, Queen’s University Belfast, 97 Lisburn Road, Northern Ireland, Belfast BT9 7BL, UK; 5 Department of Nuclear Medicine, Clínica Universidad de Navarra, Pamplona 31008, Spain; gquinfer@unav.es (G.Q.); ipenuelas@unav.es (I.P.); 6 Department of Pharmacy and Pharmaceutical Technology, University of Navarra, 31008 Pamplona, Spain

**Keywords:** Vaccine, outer membrane vesicles, *Shigella*, Intradermal, Dissolving Microneedles

## Abstract

Intradermal (ID) immunization is of increasing interest due to the easy accessibility and excellent immunogenic properties of the skin. Among ID immunization methods, dissolving microneedles (MNs) have appeared as an alternative to traditional hypodermic immunization, offering many advantages, such as being an easily administered method, with no need for health personnel, painless, and avoiding the use of needles and sharp wastage. In this study, an affordable and easy-to-produce MNs method was developed based on aqueous blends of 30% w/w poly (methyl vinyl ether-co-maleic anhydride). As an antigen model, a subunit vaccine candidate based on outer membrane vesicles from *Shigella flexneri* was used. Both unloaded and antigen-loaded MNs were synthetized and characterized. The MNs were successfully validated in an in vitro Parafilm M^®^ skin model and in a pig skin ex vivo model. Biodistribution studies were performed in BALB/c mice using ^99m^TcO_4_^−^ radiolabeled samples. Results indicated that the vesicle vaccine was successfully released from the MNs and targeted gastrointestinal tract after 6 h post-administration. In vivo immunization and protection studies were performed in BALB/c mice. Mice were intradermally immunized through ear skin with one single dose of 200 μg antigenic complex, eliciting the production of specific systemic IgG and mucosal IgA. Moreover, MNs were able to protect mice from an experimental infection with 1×10^6^ CFU/mouse of *S. flexneri* four weeks after immunization. This work demonstrates for the first time the potential of outer membrane vesicle-loaded dissolving MNs for ID vaccination against enteropathogens like *Shigella*.

## 1. Introduction

Vaccination is one of the main strategies to prevent and control infectious diseases [1]. However, during recent decades, many efforts have been being made to obtain alternatives to conventional intramuscular (IM) or subcutaneous (SC) vaccine administration routes, which are mainly administered via hypodermic needles and need trained healthcare personnel [2]. The intradermal (ID) immunization route is of great interest, due to the easy accessibility to the large surface area of the skin and because it has unique immunological properties. The skin contains a rich skin-associated lymphoid tissue (SALT), formed by a great network of immune cells, including Langerhans cells (LC), lymphocytes, and dendritic cells (DCs), which recognize the antigens and present them in the proximal lymph nodes [3]. Different ID vaccination methods have been used already, such as the Mantoux technique, microinjection systems, or jet injectors, used for smallpox, rabies, or Bacillus Calmette–Guérin (BCG) vaccines. However, they require the use of needles [4].

Microneedle patches (MNs) have appeared as a novel and attractive approach for ID immunization [5]. They are painless devices formed by arrays of micron-size projections that can pass through the stratum corneum and epidermis without reaching the nerves of the dermis layer [6]. Thus, they are a self-administered, minimally invasive method, well tolerated by human patients in proof of concept trials [7,8], that can directly target the skin immune cells [9].

Several types of MNs have been developed for the delivery of antigens: solid, coated, hollow, and dissolving MNs [10]. Among them, and based on their self-disabling properties, dissolving MNs (MNs) have been selected for this work. These MNs are made of a soluble matrix, generally a biocompatible polymer or polysaccharide, which includes the antigenic complex. After the array insertion into the skin, the needle tips dissolve and the antigenic cargo is delivered upon dissolution without generating biohazard residues [11]. Several works have now been published with dissolving MNs for vaccination purposes, among them, different antigens, such as tetanus and diphtheria toxoids or influenza hemagglutinin, have been formulated into dissolving MNs [12] and a phase I clinical trial of inactivated influenza vaccine delivered by dissolving MNs patches has recently demonstrated both immunogenicity and safety in humans [13]. Soluble protein antigens, such as toxoids, can be readily incorporated in the MNs matrix, however, the delivery of amphipathic structures is not so widely studied. To our knowledge, there is only one previous publication studying the potential of dissolving MNs for the delivery of non-toxoid bacterial antigens [14]. However, it was considered only as a basic proof of concept, since studies were not carried out to elucidate the mechanism involved.

The aim of the present study was to design and develop a dissolving MN patch made of aqueous blends of w/w poly (methyl vinyl ether-co-maleic anhydride), commercialized as Gantrez AN^®^ (Gz), which is not cytotoxic and has been shown to posses immunoadjuvant properties [15,16] and evaluate its capacity for ID vaccination. For that purpose, and based on previous reports by our group [17,18], a subunit vaccine based on outer membrane vesicles from *Shigella flexneri* was introduced into dissolving MNs as an antigens model. The mechanical and dissolving properties of MNs, as well as their immunogenic and protective capacity in mice, were evaluated. Since there is not a current licensed vaccine against shigellosis, this formulation is a promising candidate as a vaccine delivery system which could allow an easy and widespread immunization in developing countries.

## 2. Material and Methods

### 2.1. Outer Membrane Vesicle Obtention

An antigenic complex based on outer membrane vesicles was obtained from the *S. flexneri ΔtolR* mutant strain, as previously described [18]. The parental strain *S. flexneri* 2a is a clinical isolate (“Clínica Universidad de Navarra”, Spain). Both the wildtype and the mutant strains were cultured on tryptone soy agar (TSA, Biomerieux, France) or in tryptic soy broth (TSB, Biomerieux, Spain). Incubations were performed at 37 °C with shaking (140 rpm) to log phase (OD_600_∼0.3). Bacterial cultures were heat inactivated (HT) by flowing steam (100 °C) for 15 min. After centrifugation at 6 000 × *g* for 20 min, the HT-Δ*tolR* containing supernatants were filtered (0.22 µm) and purified using 100 kDa-tangential filtration (Millipore). The retenant was then harvested and centrifugated (51,000 × *g*, 1 h), and the pellets resuspended in deionized water, lyophilized, and stored at room temperature (RT) until use. The HT-Δ*tolR* antigenic complex was incorporated to MN, as described in Section 2.2.

### 2.2. Polymeric MN Arrays Formulation

Although the polymer used for this work was Gantrez^®^ AN119, other Gantrez^®^ polymers were also studied to compare efficacy and toxicity. Briefly, each microneedle (MN) was prepared from an aqueous blend of a copolymer of methyl-vinyl-ether-co-maleic anhydride (Gantrez^®^). Briefly, a stock of each polymer was prepared and then diluted with an aqueous suspension containing the HT-Δ*tolR*-based antigenic complex, to reach a determined final concentration of the polymers (Table 1). Once the formulations were homogeneous, they were centrifuged (3500 rpm, 15 min) in order to remove air bubbles.

To make the microneedle (MN) arrays, one hundred mg of the formulation containing 200 μg of the antigens was poured into each silicon micromold, avoiding bubbles, and centrifuged at 3500 rpm during 15 min to lower the formulation into the mold [19,20]. Finally, MNs were dried at room temperature (RT) for 48 h and removed from the molds. Each mold contained 361 (19 × 19) pyramidal shaped micro-needles, 500 µm tall and 300 µm wide.

### 2.3. Mechanical Characterization of MNs

#### 2.3.1. Compression Test

In order to study the mechanical properties of the MNs after application, the compression of the needles after applying a determined force [21] was studied. For this purpose, MNs were attached to the probe of a TA.XTPlus Texture Analyser (Stable MicroSystems, Surrey, UK) and the probe was lowered vertically at 1.19 mm s^−1^ until the required force of 32 N/array was applied during 30 s. MNs were visually compared before and after the compression test using a light microscope (GXMGE-5 digital microscope, Laboratory Analysis Ltd, Devon, UK), and the heights of individual needles were measured using ImageJ^®^ software. Finally, the percentage (%) of change in MN height was calculated using Equation (1), where HBC is the needle height before the compression and HAC is the needle height after the compression:

(1) % Compression =[(HBC−HAC)/HBC] ×100.

#### 2.3.2. Insertion Test

The insertion test was performed using the previously described Parafilm M^®^ method [21]. Briefly, eight layers of 1 cm^2^ Parafilm M^®^ were put on top of each other, forming a 1 mm thick layer. The Texture Analyser (TA) insertion was then performed in compression mode, as previously described. MNs were then removed from the Parafilm and each layer was unfolded. The number of holes created in each layer were counted using a Leica EZ4-D digital microscope (Leica, Wetzlar, Germany) as a measure of insertion depth.

### 2.4. Skin Insertion Studies

Ex vivo studies in skin were performed in order to confirm MN insertion and to determine the time of dissolution to establish the optimal application time for further in vivo studies. Full thickness neonatal porcine skin can be considered a good model for human skin in terms of hair sparseness and physical properties [22]. It was obtained from stillborn piglets and excised <24.0 h after birth. MNs were inserted directly in the skin for 30 s, applying a 32 N force, and the insertion was analyzed at 0, 5, 15, 30, and 60 min by optical coherence tomography (OCT), using an EX1301 VivoSight^®^ OCT Microscope (Michelson Diagnostics Ltd., Kent, UK) with a hand-held probe. Once insertion studies were performed in pig skin, studies in murine ears were carried out in order to characterize the insertion at the place of administration. Briefly, mice (*n* = 3) were sacrificed, and ears were removed and incubated at 37 °C till use. MNs were then inserted, applying pressure for 30 s. MNs were observed at time 0, 5, 15, 30, and 60 min, using OCT.

### 2.5. Immunization and Protection In Vivo Studies

All mice used in these studies were treated in accordance with institutional guidelines for treatment of animals (Ethical Committee for Animal Experimentation of the University of Navarra, Spain; Protocol ref. CEEA 164/14).

#### 2.5.1. Comparative Study between Gantrez^®^ Polymers

In order to determine the suitability of three different Gantrez^®^ polymers, nine-week-old female BALB/c mice (20 ± 1 g) were separated into seven randomized groups of six animals and were immunized with the empty or the antigen-loaded MNs formulated with different Gantrez^®^ polymers (S97, AN139 and AN119) containing 200 μg of HT-Δ*tolR* antigens. Non-immunized mice were used as control.

MNs were manually applied on the dorsal surface of the ear of the mouse under mild anesthesia, applying pressure for 5 min, and were removed after 24 h. Blood was taken before immunization (time 0) and at week 1, 2, 3, and 4 post-immunization. Total IgG or specific IgG1, IgG2a, IgG3 antibodies in sera, and IgA antibody in feces against HT-Δ*tolR* were determined by ELISA. Briefly, 96-well plates (MaxiSorb; Nunc, Germany) were coated with HT-Δ*tolR* (10 μg/well) in coating buffer (60 mM carbonate buffer, pH 9.6). Unspecific binding sites were blocked with 3% bovine serum albumin (BSA) in phosphate buffered saline (PBS) for 1 h at RT. Sera from the immunized mice were diluted 1:100 in PBS with 1% BSA and incubated for 4 h at RT. After washing with PBS Tween20 (PBS-T) buffer, class-specific goat anti-mouse conjugated antibodies were added and incubated for 1 h at RT. Absorbance was measured with an ELISA plate reader (Sunrise; Tecan-Austria, O.D. 405 nm) after incubation with H2O2-ABTS substrate-chromogen for 15 min at RT.

Four weeks after immunization, all groups were challenged with 2 × 10^6^ colony forming units (CFU) per mice of *Shigella flexneri* intranasally administered. The doses were confirmed by viable count in TSB. Two days after the infection, mice were sacrificed, and lungs were harvested for colony counting.

#### 2.5.2. Immunogenicity and Protection with Gantrez^®^ AN119

Gantrez^®^ AN119 was selected to perform further mouse experiments. Briefly, nine-week-old female BALB/c mice (20 ± 1 g) were separated in randomized groups of six animals and immunized with empty or antigen-loaded Gantrez^®^ AN119 MNs. With the aim of comparing two different doses of HT-Δ*tolR* in MNs, a low concentration (20 μg/mouse) and a high concentration (200 μg/mouse) of antigens were tested. A group immunized with one single intranasal dose of HT-Δ*tolR* (20 μg) and a non-immunized group were used as controls. Blood was taken before immunization (time 0) and at week 1, 2, 3, and 4 post-immunization. Specific serum IgG1, IgG2a, and fecal IgA antibodies against HT-Δ*tolR* were determined by ELISA, as previously described. Four weeks after immunization, all groups were challenged intranasally with 2 × 10^6^ CFU of *S. flexneri* per mouse. Two days after the infection, mice were sacrificed, and lungs were harvested for colony counting.

### 2.6. Biodistribution Studies

Radiolabeling of HT-Δ*tolR* was performed by technetium-99m (^99m^TcO_4_^−^) reduction with Sn^++^. Sodium pertechnetate was obtained by elution of a ^99^Mo-^99m^Tc generator (10 GBq Drytec, General Electric) following the manufacturer’s instructions. Forty µL of SnCl_2_·2H_2_O (1 mg/mL) was used and no ^99m^Tc-tin colloids were produced during the radiolabeling reaction. HT-Δ*tolR* (300 mg) were pre-tinned with a HCl acidified tin chloride solution, ^99m^TcO_4_^−^ in saline added and reduction carried out in a non-oxidizing atmosphere using He-purged vials and solutions. The radiochemical purity of radiolabeled vesicles was checked by thin layer chromatography (TLC) using Whatmann 3MM strips (GE Healthcare, Chicago, IL, USA) developed with 0.9% NaCl. Radioactivity distribution was measured and quantified using a radioTLC system (miniGITA, Raystest GmBH, Straubenhardt, Germany). Radiolabeling proceeded with >95–97% yield, thus avoiding the need for further purification of the radiolabeled product. After the quality control, 146 L of the radiolabeled vesicles was mixed with 439 mg of Gantrez^®^ AN 119 (40%) to reach a concentration of 0.44 μg radiolabeled HT-Δ*tolR*/mg formulation in a 30% Gantrez^®^ solution. Finally, MNs were fabricated as mentioned above (see Section 2.1), obtaining 20 μg of radiolabeled HT-Δ*tolR*/patch. MNs were manually applied on the dorsal surface of the ear of the mouse under mild anesthesia and removed after 6 h. The selection of this period of time was due to the half-life of the ^99m^Tc selected for this study, which is 6 h, and hence any activity dose would have dropped after that period of time [23].

### 2.7. Statistical Analysis

Statistical analysis was carried out using parametric t-student test, one-way ANOVA, or the non-parametric Mann-Whitney U test, as required. *p* values of <0.05 were considered statistically significant. All calculations were performed using GraphPad Prism5^®^ software.

## 3. Results

### 3.1. Polymeric MN Arrays Formulation

**Figure 1:**
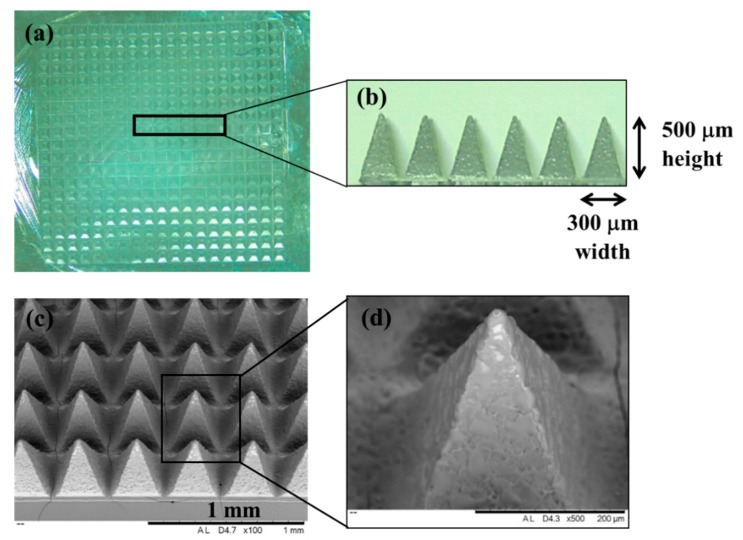
Representative images of Gantrez^®^ AN119 dissolving microneedles (MNs) arrays. (**a**) Digital images of a complete MN array 500 μm in height and 300 μm in width and (**b**) single row of the array. (**c**) Scanning electron microscopy image of an array section with pyramidal shape, and (**d**) individual needle with sharp tip.

The obtained Gantrez^®^ based MNs were 500 µm high and 300 µm wide at the base, containing 19 × 19 pyramidal shaped needles with sharp tips, ideal for insertion into the skin (Figure 1). The different Gantrez^®^ polymers were able to form complete arrays of needles, and the antigens did not seem to affect the structural properties of the MNs.

### 3.2. Mechanical Characterization of MNs

**Figure 2:**
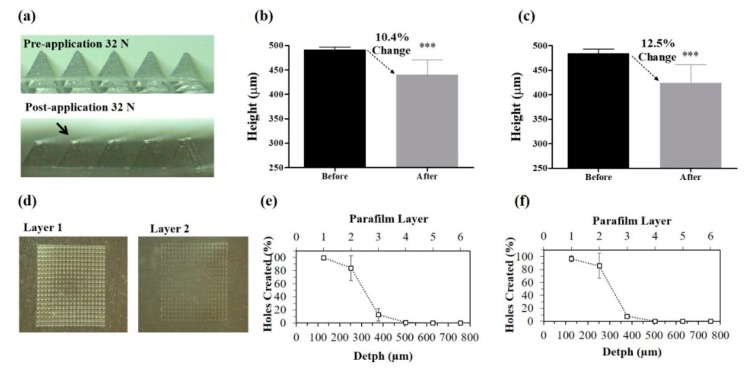
Mechanical characterization of dissolving empty or antigen-loaded Gantrez^®^ AN119 MN in murine skin. (**a**) Height of MNs pre and post application of MN with a force of 32 N and percentage of change of (**b**) empty-MN or (**c**) HT-Δ*tolR*-loaded MNs (***, *p* < 0.001). (**d**) Parafilm layers showing the holes created after the insertion of the MN and the percentage of holes in different layers correlated with the depth of insertion of (**e**) empty-MN or (**f**) HT-Δ*tolR*-loaded MNs.

In order to characterize the mechanical properties of Gantrez^®^ AN119 MNs, arrays were submitted to a force of 32 N, previously stablished by Larrañeta et al., as the mean force that is exerted on the skin by humans when applying the patches [21], and their compression and insertion capacities were evaluated. After the application of the force, both empty and loaded-MNs decreased in height (10.4% and 12.5%, respectively), showing no significant differences between them, thus demonstrating that antigens did not affect the integrity of the patch (Figure 2a–c).

Moreover, both empty and loaded-MNs could successfully insert in Parafilm M^®^ (Sigma-Aldrich, St. Louis, MO, USA), obtaining a similar percentage of holes created in each layer, with 80% of the needle tips inserted through the first two layers. Moreover, less than 20% of the needle tips were capable of being inserted through the third and fourth layers. Thus, the MN insertion depth was between 300 μm and 400 μm, which represents approximately 60–80% of the needle length (Figure 2d–f).

### 3.3. Skin Insertion and Dissolution Studies

#### 3.3.1. Insertion in Pig Skin

**Figure 3:**
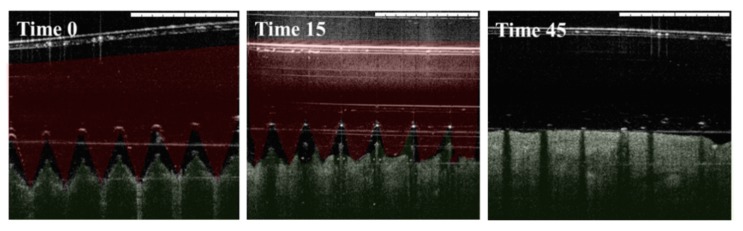
Optical coherence tomographic images of dissolving Gantrez^®^ AN119 MN in porcine skin. Images show in vitro dissolution kinetics at time 0, 15, and 45 min after insertion in porcine skin. Red color represents needles and green color skin. Scale bars: 1 mm.

Ex vivo insertion studies were first performed in porcine skin, as a good model for human skin [22], and were visualized using optical coherence tomography (OTC). Both empty and antigen-loaded MN arrays could successfully insert into porcine skin. As observed in Figure 3, MN array started to dissolve after the application, and was completely dissolved after 45 min.

#### 3.3.2. Insertion in Mice Skin

**Figure 4:**
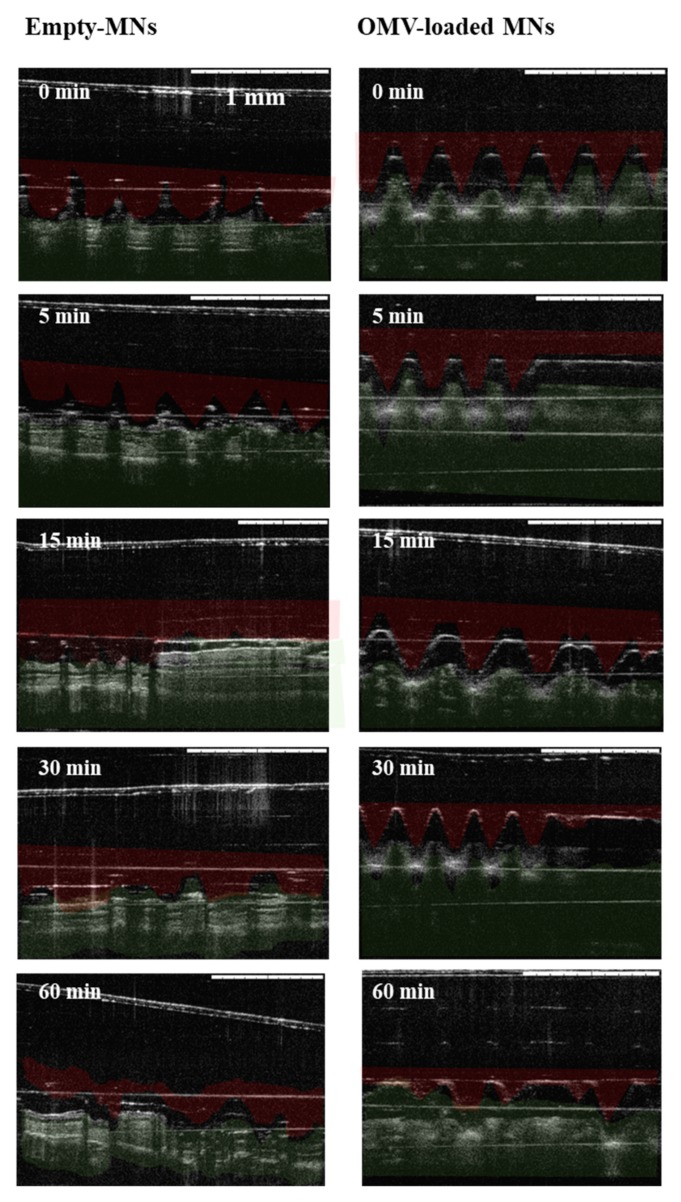
Optical coherence tomographic (OTC) images of dissolving empty or antigen-loaded MN in murine skin. Images show in vitro dissolution kinetics at 0, 5, 15, 30, and 60 min after insertion in murine ear. Red color represents needles and green color skin. Scale bars: 1 mm.

After the ex vivo studies in porcine skin, insertion was evaluated in murine ear skin, since it was the place of administration in further in vivo studies, and images were also analyzed by OTC. As observed in Figure 4, both empty and antigen-loaded MN arrays could also insert into mice skin, and different images showed that 60 min after the application of MNs, the MNs were almost completely dissolved.

### 3.4. Immunization and Protection Studies In Vivo

**Figure 5:**
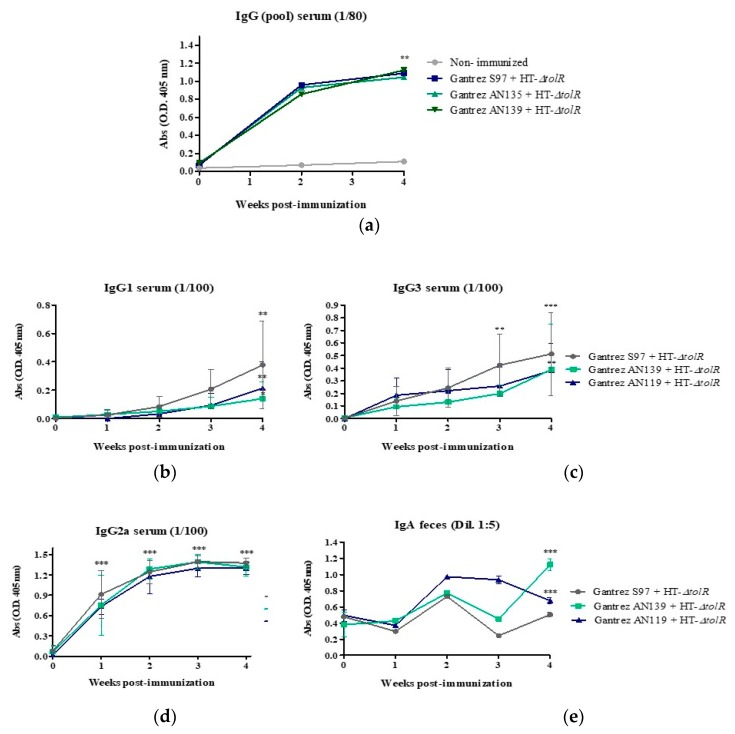
Antibody immune response induced after skin vaccination of mice with HT-Δ*tolR* or embedded in Gantrez^®^ AN119 microneedles. Specific serum IgG (**a**), IgG1 (**b**), IgG3 (**c**), and Ig2a (**d**) levels against HT-Δ*tolR*, as well as IgA (**e**) levels in feces of immunized BALB/c mice. Blood and fecal samples were taken from weeks 0 to week 4 after immunization (**, *p* < 0.01, ***, *p* < 0.01 versus time 0). Error bars represent SD (*n* = 6).

In a first animal study, mice were immunized with MNs formulated with different Gantrez^®^ polymers (S97, AN139 and AN119) containing 200 μg of HT-Δ*tolR* antigens. Blood was taken once four weeks post-immunization and specific serum levels of total IgG, IgG1, IgG2a, IgG3, and fecal IgA against HT-Δ*tolR* antigens were determined by ELISA. Results indicated that the immunization with MN elicited significant levels of serum total IgG (*p* < 0.01). Specifically, serum IgG2a and IgG3 (*p* < 0.001), were increased four weeks post-immunization with respect to time 0, whereas IgG1 levels were lower (*p* < 0.01). Finally, immunization with MNs also elicited high levels of IgA in mice feces (*p* < 0.001) (Figure 5).

**Figure 6:**
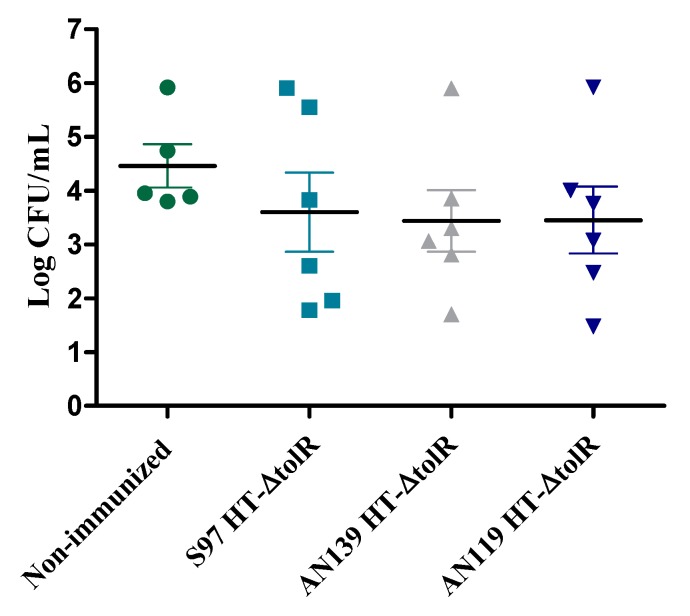
Protection against colonization of *Shigella flexneri*. Mice were immunized with one single dose of 200 μg of the antigenic complex HT-∆*tolR* embedded in in different formulations of Gantrez^®^ MNs (six mice per group). A non-immunized group was included as control. Results are presented as degree of colonization (Log CFU/mL) in lungs. Error bars represent SD.

Four weeks post-immunization, mice were infected via the intranasal route with a sublethal dose of 1 × 10^6^ CFU/mouse. Lungs were harvested and bacteria were counted. The three different groups of immunized mice showed a decrease in CFU/mL with respect to non-immunized group, although no significant differences (*p* > 0.05) were observed between the non-immunized group and the different Gantrez^®^ formulations groups (Figure 6).

Once it was confirmed that Gantrez^®^ AN119 showed no differences with respect to the other Gantrez^®^ polymers, and since this particular polymer has not been previously used before to form MNs, this polymer was selected to perform further mice experiments.

**Figure 7:**
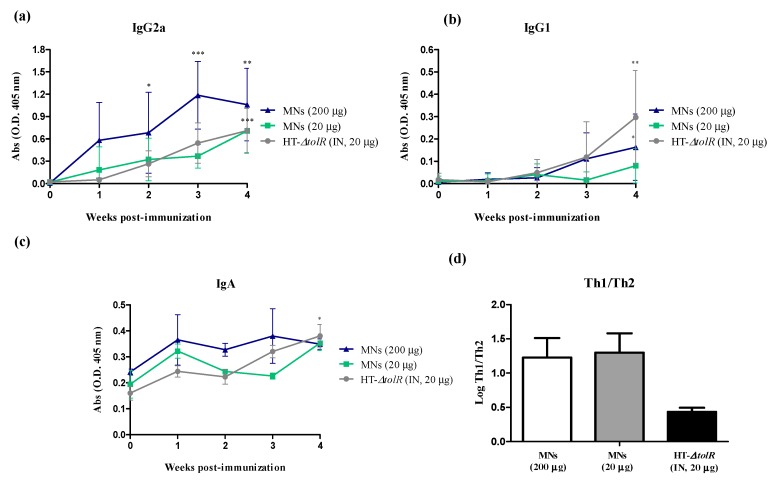
Antibody immune response induced after skin vaccination of BALB/c mice with free HT-Δ*tolR* or embedded in Gantrez^®^ AN119 microneedles. Specific serum IgG2a (**a**) and IgG1 (**b**) or fecal IgA (**c**) against HT-∆*tolR* in immunized BALB/c mice with 20 μg of HT-∆*tolR* or embedded in MNs (20 or 200 μg) (*, *p* < 0.05, **, *p* < 0.01, ***, *p* < 0.001, versus time 0). Blood and fecal samples were taken from week 0 to 4 week after immunization. The balance of Th1/Th2-immune response (**d**) is represented by the logarithm (Log) of the relative expression levels of IgG2a/IgG1 absorbance at week 4 post-immunization. Error bars represent SD.

Thus, in order to determine whether the immunization with Gantrez^®^ AN119 MNs was protective against an infection with *S. flexneri,* mice were immunized with a low (20 μg) and high (200 μg) dose of the antigens loaded in the MNs and blood was taken once per week for four weeks. Specific serum levels of IgG1 and IgG2a and fecal IgA against HT-Δ*tolR* antigens were determined by ELISA. The immunization with both MN groups and free antigens elicited significant levels of serum IgG2a after four weeks post-immunization with respect to time 0 (*p* < 0.001) (Figure 7a), whereas only mice immunized with MNs with 200 μg (*p* < 0.05) or free antigens (*p* < 0.01) elicited significant IgG1 levels (Figure 7b). This imbalanced result in the Th1 and Th2 antibody release was represented with the relative amount of IgG2a and IgG1 at week 4 post-immunization. Results indicate that the groups immunized through MNs showed a marked Th1 profile, whereas the difference between both antibodies was lower in the group immunized IN with the free antigens (Figure 7d). Regarding mucosal immune response, only mice immunized with free antigens showed significant levels of IgA in feces in week 4 with respect to time 0 (*p* < 0.05), although the levels of IgA elicited by MNs with high dose of antigens were significantly higher than this group until week 3 (*p* < 0.05) when compared between them (Figure 7c).

**Figure 8:**
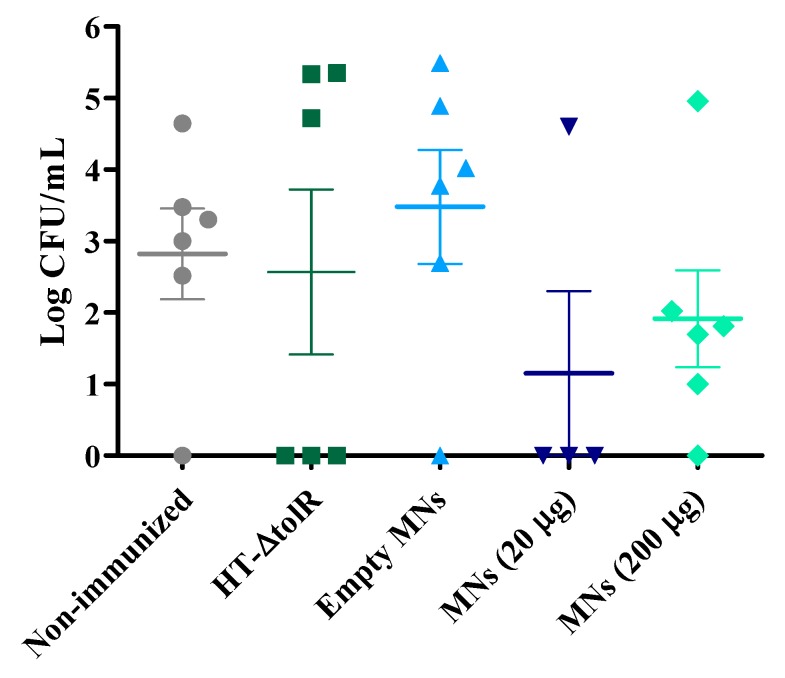
Protection against colonization of *Shigella flexneri*. Mice were immunized with one single dose of 20 μg of free HT-∆*tolR* (intranasally) or 20 or 200 μg of HT-∆*tolR* embedded in Gantrez^®^ AN119 MNs (six mice per group). Results are presented as degree of colonization (Log CFU/mL) in lungs. Non-immunized and empty-MN groups were included as controls. Error bars represent SD.

Four weeks post-immunization, mice were infected intranasally with a sublethal dose of 1 × 10^6^ CFU/mouse. Lungs were harvested and CFU were counted. Responder and non-responder mice could be identified in all immunized groups. Results showed a decrease in the number of CFU in vaccinated mice with respect to the non-immunized group. Moreover, the bacteria cargo also decreased in the two groups immunized with MNs when compared to mice immunized intranasally with the free antigens. Finally, no differences were obtained between the two different antigen doses tested in MNs (Figure 8).

### 3.5. Biodistribution Studies

**Figure 9:**
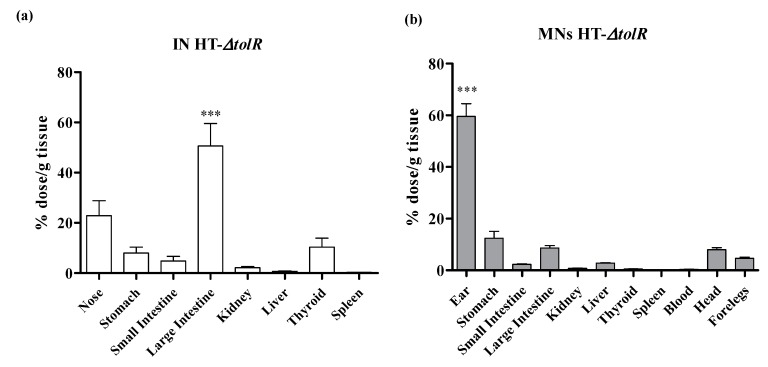
Biodistribution of radiolabeled HT-∆*tolR* after nasal or intradermal administration in BALB/C mice. Percentage (%) of ^99m^Tc activity in the different organs 6 h after (**a**) intranasal, IN or (**b**) microneedles (MN) intradermal administration of 20 µg HT-∆*tolR*. Due to the different organs evaluated in each route, results were evaluated separately. The results show the mean and SD (*n* = 5) (***, *p* < 0.001). IN: intranasal; MNs: microneedles.

In order to compare the effect of MNs on the biodistribution of the vaccine, ^99m^Tc-HT-∆*tolR* (20 µg) was administered either intradermally with MNs or intranasally, as control. After nasal administration, the percentage of radioactivity in the large intestine increased (50%) as radioactivity in the nose decreased (23%) 6 h post-administration (Figure 9a). Radiolabeled ^99m^Tc HT-∆*tolR* were not detected in the other organs studied.

After the administration of MNs on the ear skin of the mice, almost 60% of the radioactive signal remained in this location. From the amount (40%) distributed to other organs, more than half (56%) of the formulation was detected in the gastrointestinal tract 6 h post-administration (Figure 9b).

## 4. Discussion

In recent years, there has been an increasing requirement for alternative vaccine delivery systems to avoid the use of injectable devices, which need trained personnel for their administration and may require specialized storage and transport conditions [24]. This is especially important in the developing world, where numerous infectious diseases are endemic [25]. Furthermore, from an immunological point of view, although injectable vaccines are capable of eliciting robust systemic responses, they sometimes suffer from a lack of mucosal protection, which is essential to protect against specific infectious agents, such as enteropathogens [24]. The main alternative routes of vaccine delivery without needles capable of eliciting both mucosal and systemic immune responses are mucosal and intradermal ones [26]. These routes stimulate the mucosa-associated lymphoid tissue (MALT) (e.g., the gut-associated lymphoid tissue, GALT, and the nasal-associated lymphoid tissue, NALT), and the skin-associated lymphoid tissue (SALT). Moreover, these routes are attractive due to the rapid and wide biodistribution of the antigens, for being self-administered and painless, avoiding the use of needles, and not needing healthcare personnel [27].

The ID route is particularly interesting to get patients’ compliance due to the easy accessibility to the skin [28]. A number of strategies have been proposed for the ID delivery of antigens without the employment of needles, such as high-velocity needle-free injection systems, permeability enhancers to disrupt the stratum corneum, or the use of antigen delivery systems with percutaneous absorption properties or diffusive capacity [29]. Among these, microneedles (MNs) are gaining special interest due their easy and self-vaccination process and the lack of generation of biohazardous waste, avoiding problems related to needle-phobia, needle-associated infections, or waste management [30].

Among them, and based on their self-disabling properties, dissolving MNs have been selected for this work. MNs were prepared by using Gantrez^®^, a poly(methyl vinyl ether-co-maleic anhydride) polymer, which has been extensively employed for pharmaceutical applications, such as denture adhesives, thickening, suspending agents, or carriers for oral delivery, due to its mucoadhesive properties and low oral toxicity [31]. More recently, it has been used for the preparation of transdermal patches, showing good properties to form dissolving MNs [14,32]. Among the different types of Gantrez^®^ polymers used, Gantrez^®^ AN119 has not been reported to perform MNs. We have previously shown that this particular polymer is non-cytotoxic and has immunoadjuvant properties [15,16]. Indeed, AN119 MNs were successfully produced and showed excellent mechanical and insertion properties when analyzed in both Parafilm M^®^ and neonatal porcine skin (Figure 2). Moreover, OCT showed that the MNs could successfully penetrate murine skin and the microprojections of the array were completely dissolved in the first hour after application. In vivo studies in mice also demonstrated the safety of this polymer. Previous studies demonstrated that a repeated application of Gantrez^®^ MNs does not cause any sign of skin alteration in mice [33]. This may be a critical factor, since dissolving MN deposit the polymer on the skin follows complete dissolution and local irritation and erythema have been described elsewhere [10]. In the current study, mice ears did not show any significant irritation or necrotic signs, even after 24 h of application. Moreover, a single dose of the vaccine was considered, which would decrease the risk of any side effect.

MNs were loaded with HT-∆*tolR* antigenic complex, an outer membrane vesicle-based vaccine obtained from heat-inactivated *S. flexneri* ∆*tolR* strain. The complex was previously characterized and demonstrated to be immunogenic and protective in a murine model of *shigellosis* [18]. To our knowledge, this is the first time that outer membrane vesicles have been administrated through dissolving MNs. To demonstrate the efficacy of the vaccine after cutaneous administration, immunogenicity and protection were evaluated.

In a first in vivo study, a comparison between Gantrez^®^ AN119 and the polymers AN139 and S97 already used in other studies [11,14,34] was performed. Comparative immunization and protection results did not show significant differences between polymers. The three groups elicited high levels of specific antibodies and were able to protect mice against an experimental infection with S. flexneri (Figure 5 and Figure 6). These data support the use of the AN119 polymer for transcutaneous immunization, and further in vivo studies were performed. Immunization results confirmed that the antigenic complex embedded in AN119 MNs elicited a predominant IgG2a response (Figure 7), indicating a marked Th1 immunity, pro-inflammatory response which is necessary to detain *S. flexneri* infection [35]. On the contrary, when the antigenic complex was delivered in solution intranasally, the Th1/Th2 balance was significantly lower. These data suggest a possible role of the Gantrez^®^ polymer as Th1-adjuvant in the MN delivery. In fact, the role of SALT in inducing a pro-inflammatory response has been previously shown [36]. Furthermore, high levels of IgA were observed in feces from week 1 after immunization with MNs with 200 μg of the antigenic complex, and protection was achieved against an experimental infection. Mucosal secretory IgA is a critical factor to face enteropathogens in the sites of colonization [37,38]. Thus, elevated levels of specific antibodies have been associated with reduced incidence of shigellosis in naturally exposed individuals. Moreover, LPS and IpaB-specific IgA have also been proposed as indicators of protective immunity [39].

All these results indicated the penetration of the HT-∆*tolR* from the MN patch through the skin layers into the circulation, which was corroborated by the biodistribution studies performed with radiolabeled vaccine. From the vaccine formulation distributed among the different organs, almost 60% was located in the gastrointestinal tract. The connection between the skin and gut is possible through the interconnections of MALT. The great network of immune cells of the SALT recognize the antigens and migrate to peripheral lymph nodes or circulate in the blood to other tissues, including mucosae, reaching the GALT [40].

## 5. Conclusions

In summary, the HT-Δ*tolR* antigenic complex was successfully loaded into dissolving MNs for intradermal administration, eliciting specific systemic and mucosal immune responses, which were protective against an experimental infection in mice. This delivery system represents a simple, affordable, and easy-to-use method of vaccine administration against enterobacterial pathogens, facilitating immunization processes in low- and middle-income countries.

## Figures and Tables

**Table 1:** Gantrez^®^ AN polymers used in this work.

Polymers	MW (g/mol)	Solvent	Final Concentration
**Gantrez^®^ AN119**	216,000	Water	30%
**Gantrez^®^ AN139**	1,080,000	Water	20%
**Gantrez^®^ S97**	1,500,000	Water	30%

MW: Molecular weight.
